# Preoperative Three-Dimensional Planning Using Computed Tomography Improves Screw Placement in Patients Undergoing Acetabular Revision Surgery

**DOI:** 10.1016/j.artd.2024.101431

**Published:** 2024-09-18

**Authors:** Jonathan Brandt, Rolf Scheiderbauer, Daphne Wezenberg, Jörg Schilcher

**Affiliations:** a Department of Orthopaedic Surgery, Capio Specialistvård Motala, Motala, Sweden; b Department of Orthopaedic Surgery and Department of Clinical and Experimental Medicine, Faculty of Health Sciences, Linköping University, Linköping, Sweden; c Sectra Orthopaedics AB, Linköping, Sweden; d Wallenberg Centre for Molecular Medicine, Linköping University, Linköping, Sweden; e Center for Medical Image Science and Visualization (CMIV), Linköping University, Linköping, Sweden

**Keywords:** Hip replacement, Revision surgery, Screw fixation, Preoperative planning, Acetabular cup placement

## Abstract

**Background:**

Stable fixation of joint replacement implants is essential to achieve osseointegration in uncemented implants. In acetabular revisions, screws often need to be utilized in quadrants other than the historically so-called “safe” zones to attain sufficient stability. The primary aim of this study was to determine whether preoperative three-dimensional (3D) planning for acetabular revision surgery influences screw length, specifically in the superior pubic ramus (SPR).

**Methods:**

Between March 2017 and December 2021, 20 patients underwent preoperative two-dimensional (2D) planning (*2D group*), and 30 patients underwent 3D planning following the implementation of a new 3D planning software into clinical practice in September 2019 (*3D group*). Two observers, blinded to the groups, measured the *total screw length*, *screw penetration depth*, and cup position on available postoperative computed tomography examinations. For statistical comparisons, the mean measurement from the 2 observers was used.

**Results:**

The median *total screw lengths* in the SPR were 16 mm in the *2D group* and 25 mm in the *3D group* (*P* = .004) and 40.5 mm compared with 50.5 mm in the ilium (*P* = .019). Median *screw penetration depths* in the SPR were 0 mm in the *2D group* and 1.25 mm in the *3D group* (*P* = .049).

**Conclusion:**

Longer screws were used in the SPR and ilium when preoperative 3D planning was conducted. Due to the study design, we were not able to evaluate whether longer screws lead to better fixation. Further studies are needed to elucidate this question.

## Introduction

Acetabular revision surgery can be challenging due to bone defects requiring the use of a bone graft or augmentation with metal constructs [1,2]. Bone grafting offers the advantage of bone-stock restoration, facilitating further revisions if needed [3]. Metal constructs, including augments or custom-made implants, are designed to ensure sufficient contact with the host bone to adhere with the 50% host-bone paradigm [4,5]. This paradigm builds upon the idea that insufficient contact with the host bone hampers osseointegration. However, severe bone defects also affect mechanical stability, jeopardizing osseointegration [6]. Screw fixation in uncemented revision implant surgery might improve implant stability such that osseointegration can occur even without fulfilling the 50% host-bone requirement [7]. One downside of acetabular screw fixation is the risk of damaging the surrounding anatomic structures [8,9]. Placing screws in the posterior superior and posterior inferior zones of the acetabulum is generally considered safe, but the anterior superior and anterior inferior zones should be avoided to not damage the iliac or obturator artery or vein and ischial nerve [8,10]. However, when implant stability relies on screw fixation, screw placement becomes crucial to achieve implant stability. In these situations, the benefits associated with screw placement need to be balanced against the risks associated with poor implant stability or the need for additional, extensive reconstructions such as custom-made implants [11,12].

High-quality computed tomography (CT) imaging combined with modern three-dimensional (3D) visualization techniques enables the surgeon to analyze the bony patho-anatomy, to evaluate different reconstruction techniques including screw trajectory and length, before the surgery [13].

In this study, we aimed to investigate acetabular revisions utilizing traditional 2D planning compared with 3D planning and the effect of these different visualization techniques on screw length, specifically in the SPR. We hypothesized that 3D planning promotes longer screws.

## Material and methods

This study was performed at Linköping University Hospital, a tertiary referral center for complex hip arthroplasty cases for the South Eastern Health Care District of Sweden, serving roughly 1.1 million people [14].

### Patients

According to the Swedish Joint Arthroplasty Registry (data extracted March 2, 2022; criteria: KVÅ-code NFC20-99), 82 acetabular revisions were performed at our hospital between March 2017 and December 2021. Patients with multiple reoperations (*N* = 5) were only included for their first reoperation. In September 2019, we implemented routine 3D planning for most of our acetabular revision surgeries. Patients operated before that date comprised the *2D group*, and those operated after and where 3D planning was used comprised the *3D group*. According to the institutional protocol, a digital template of the surgical plan was saved in the Picture Archiving and Communication System system for 3D-review during surgery.

**Figure 1:**
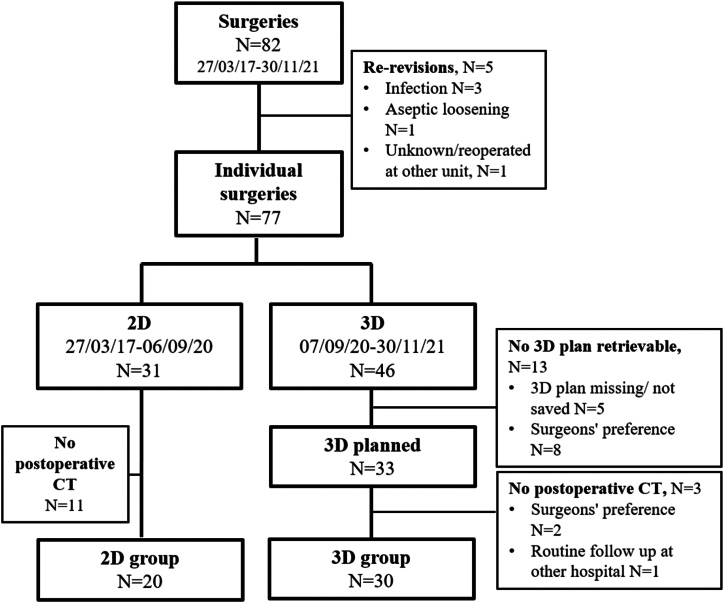
Flowchart of the study population. The 8 patients who were planned in 2D during the 3D period were excluded from the analysis.

Some patients were excluded from the *3D group* because the surgical 3D plan was not available at the time of review (*N* = 5), or the preoperative planning was done in 2D during the 3D period based on surgeon preference (*N* = 8). Patients where no postoperative CT of the revised hip was available (*N* = 11 in the *2D group*, *N* = 3 in the *3D group*) were excluded. The final cohort comprised a total of 50 patients: 20 patients in the *2D group* and 30 patients in the *3D group* (Fig. 1, Table 1).

**Table 1:** Clinicopathologic characteristics of the patients in the 2 groups.

Characteristic	2D	3D	*P*-value
Age [IQR]	70.0 [64.0-74.5]	70.0 [59.5-77.8]	.905
Sex (%)			1.000
Women	11 (55.0)	16 (53.3)	
BMI (SD)	28.1 (5.51)	28.1 (4.69)	.987
AAOS classification (%)			.835
0	2 (10.0)	1 (3.33)	
1	3 (15.0)	6 (20.0)	
2	3 (15.0)	5 (16.7)	
3	12 (60.0)	18 (60.0)	
ASA (%)			.315
I	1 (5.00)	3 (10.0)	
II	14 (70.0)	13 (43.3)	
III	5 (25.0)	12 (40.0)	
IV	0 (0.00)	2 (6.67)	
Number of previous revisions (%)			.182
0	10 (50.0)	22 (73.3)	
1	3 (15.0)	3 (10.0)	
2	3 (15.0)	4 (13.3)	
3	3 (15.0)	0 (0.00)	
4	1 (5.00)	1 (3.33)	
Stem revised (%)	7 (35.0)	16 (53.3)	.163
Revision cup			.349
Trident Tritanium	18	29	
Trident HA Multihole	2	1	
Size revision cup in mm [IQR]	60 [52.0-58.0]	60 [53.0-67.0]	.39

AAOS, American Academy of Orthopedic Surgeons Classification of Acetabular Deficiencies; ASA, American Society of Anaesthesiologists physical status classification system; BMI, body mass index; IQR, interquartile range.

### Implants

All surgeries, except 3, were performed using the Trident Tritanium-Acetabular System (Stryker Orthopedics, Mahwah, NJ). The remaining 3 patients were operated with the Trident HA Multihole-shell (Stryker Orthopedics, Mahwah, NJ).

### Preoperative planning

**Figure 2:**
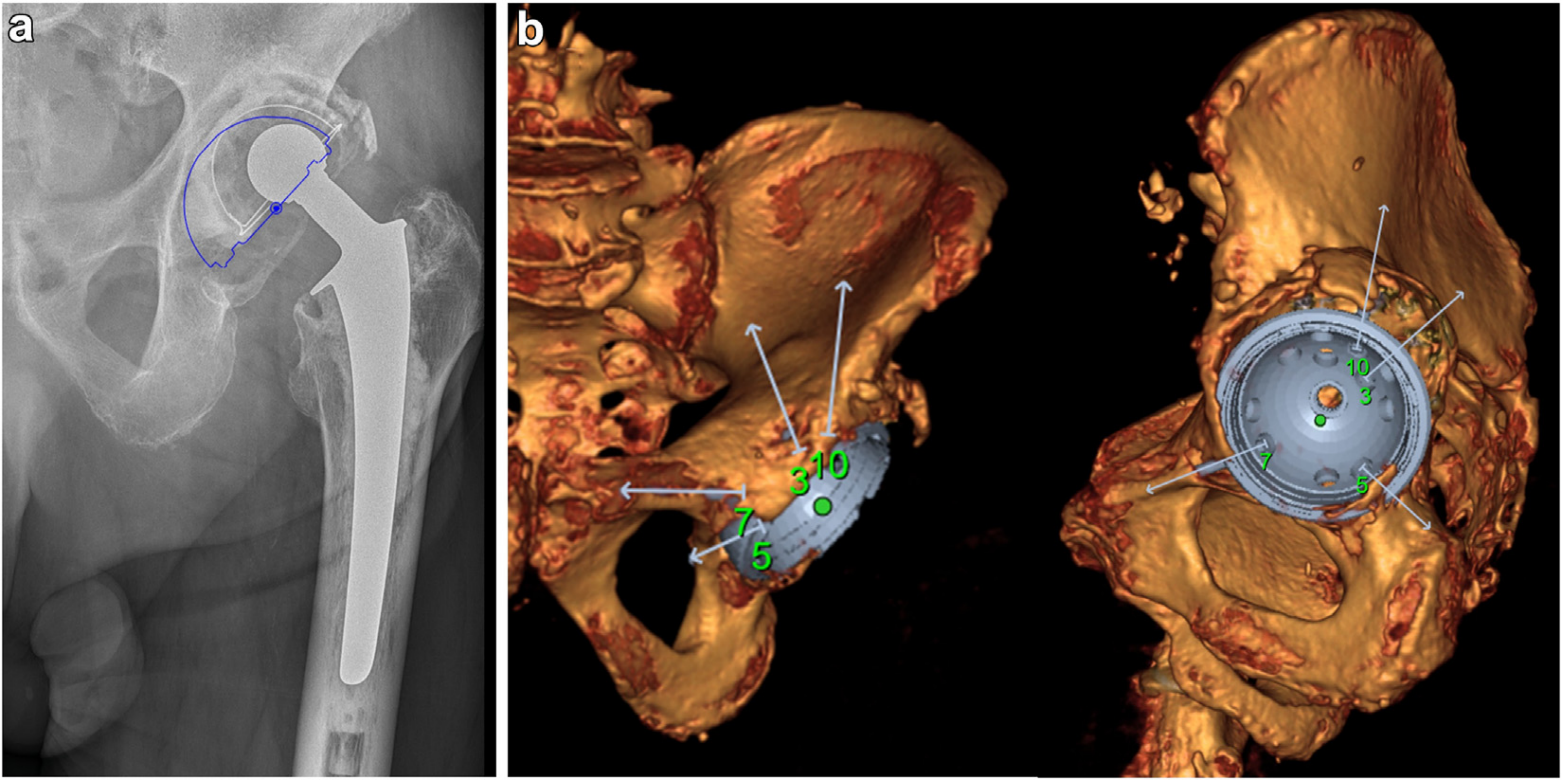
(a) Traditional 2D planning. (b) A 3D model of the pelvis and the implant with screw trajectory simulation (gray arrows).

All planning was performed in Sectra IDS7 (Sectra AB, Linköping, Sweden), using Sectra Orthostation for 2D planning and Sectra 3D Joint Replacement for 3D planning. For 2D planning, the surgeon used a calibrated plain x-ray of the pelvis and either measured the size of the existing implant/bone defect or placed a digital template of the cup over the existing implant (Fig. 2a). For 3D planning, a high-quality CT with metal artifact reduction protocol was used to create a 3D model of the pelvis, to visualize the revision in all dimensions (Fig. 2b). A digital template of the cup was then placed in position based on available host bone; surgeon preference; and predicted likelihood of screw placement achieving a stable reconstruction. Screw placement was simulated through available screw holes with angular restriction based on the manufacturer´s specification (see Supplementary Video Material). Due to the often-poor bone quality in the ischium and SPR, we planned for these screws to penetrate the far cortex, so as to improve screw purchase [15]. Special care was taken to reconstruct the anatomic center of rotation of the hip. Placement of the cup in its anatomic position ensures screw fixation in the SPR and the ischium. A high hip center of rotation precludes such screw placement [16,17].

### Surgeries

**Figure 3:**
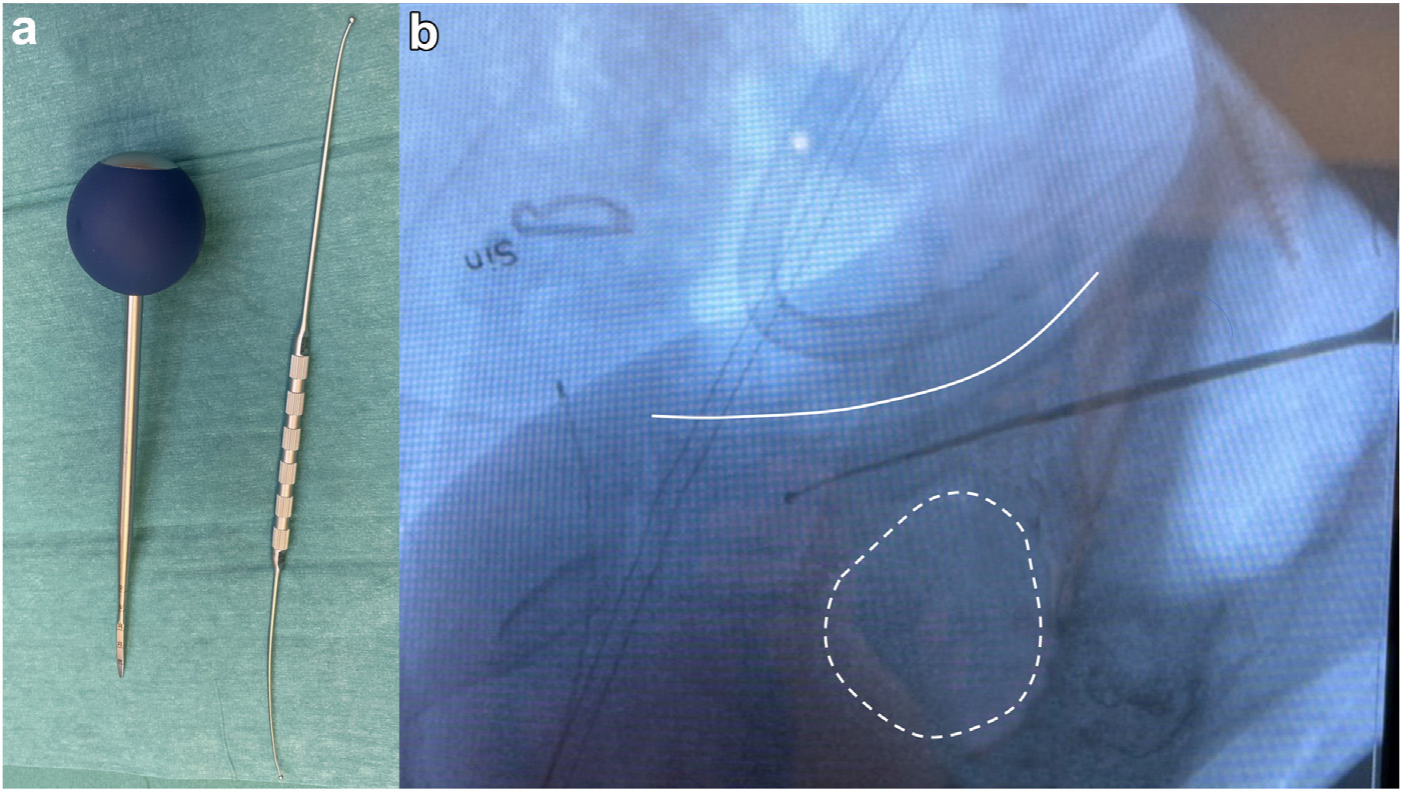
(a) A curved thoracic pedicular *finder* (left) and a *feeler* (right) help to identify the superior pubic ramus. (b) Perioperative fluoroscopy image depicting a spinal pedicular *feeler* placed in the superior pubic ramus. The solid white line indicates the superior aspect of the superior pubic ramus, and the dotted line indicates the margins of the obturator foramen.

All surgeries were performed by 3 senior orthopedic surgeons specialized in hip revision surgery, using a posterior approach and standardized bone-grafting protocol [3]. Briefly, the acetabulum was prepared with hemispherical reamers before trialing with the smallest acetabular shell that allowed press-fit contact with the host bone (when possible) between at least 2 opposing portions of the remaining acetabular wall. The final implant was seated in the same position, and screw fixation was performed irrespective of the perceived stability of the implant. In the *2D group*, drilling for the SPR screw was based on anatomical landmarks and, if possible, palpation of the SPR through a fenestration in the anterior capsule. In the *3D group*, the digital template was used to help identify the entrance point and angulation of the drill. In both groups, the digital preoperative plan was available on a large screen in the operating room to assist the surgeon in identifying anatomical landmarks from the preoperative plan and to reproduce screw trajectories during surgery. A flexible depth gauge was used to measure screw length, and in some cases, a spinal pedicular finder and feeler were used to identify the bony canals and estimate screw length (Fig. 3) [18]. The first screws were typically inserted into the inferior parts of the shell to prevent tilting in abduction, followed by the insertion of screws in the superior part of the shell. In some cases, structural allografts (*N* = 1), metal augments (*N* = 2), or meshes (*N* = 2) were used to facilitate containment of either the cup or the bone graft. All patients entered rehabilitation including early mobilization with partial or full weight-bearing for 6 weeks.

### Outcome measures

#### Primary

The primary outcome measure was the difference in *total screw length* in the SPR between the groups.

#### Secondary

Secondary outcomes were the difference in *screw penetration depth* of the screws in the SPR; *total screw length* and *screw penetration depth* of screws in the ilium and ischium; cup position (*inclination* and *anteversion*); rotational center of the revision hip in relation to the contralateral side; and the total number of screws. We also examined differences in clinical outcome measures using the Merle d'Aubigné-Postel score, preoperatively and at 1 year postoperatively [19,20].

### Evaluation of screw position and length

**Figure 4:**
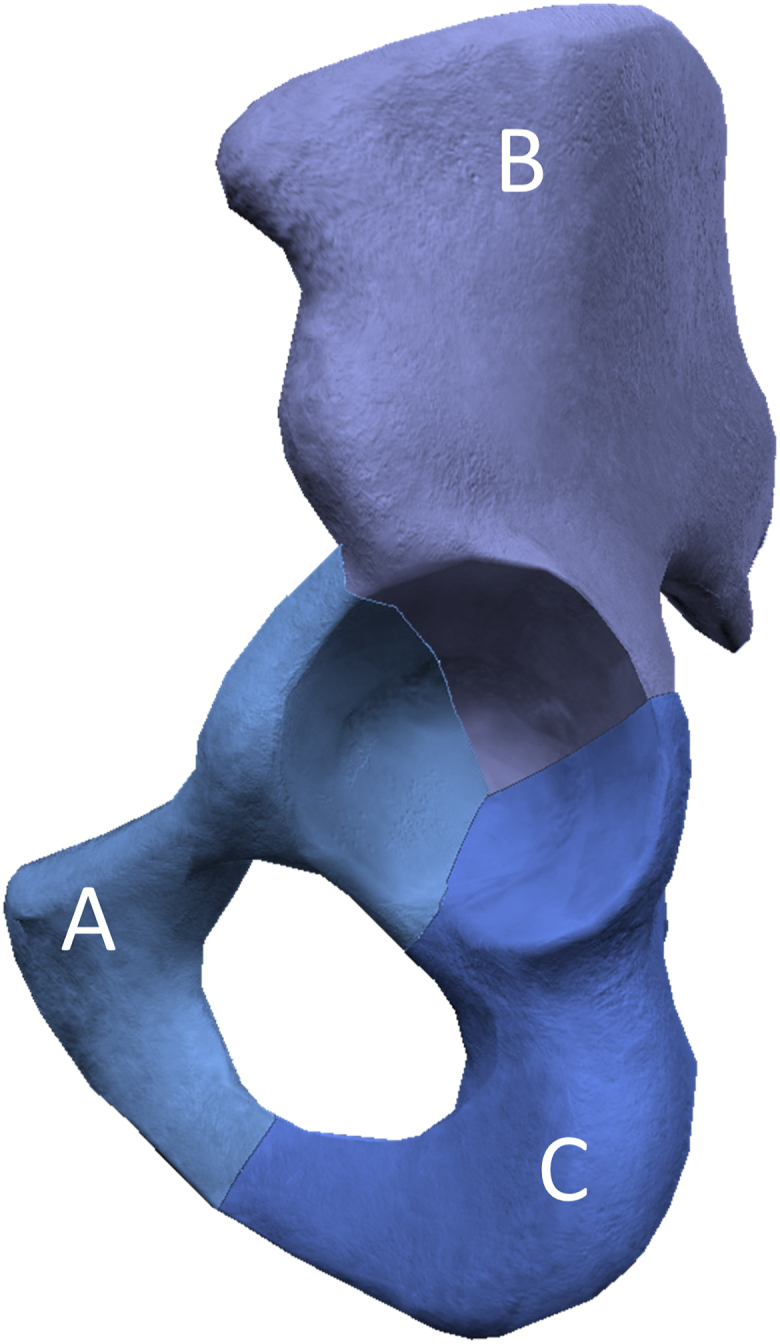
The screws are categorized according to anatomic region: superior pubic ramus (A); ilium (B); and ischium (C).

**Figure 5:**
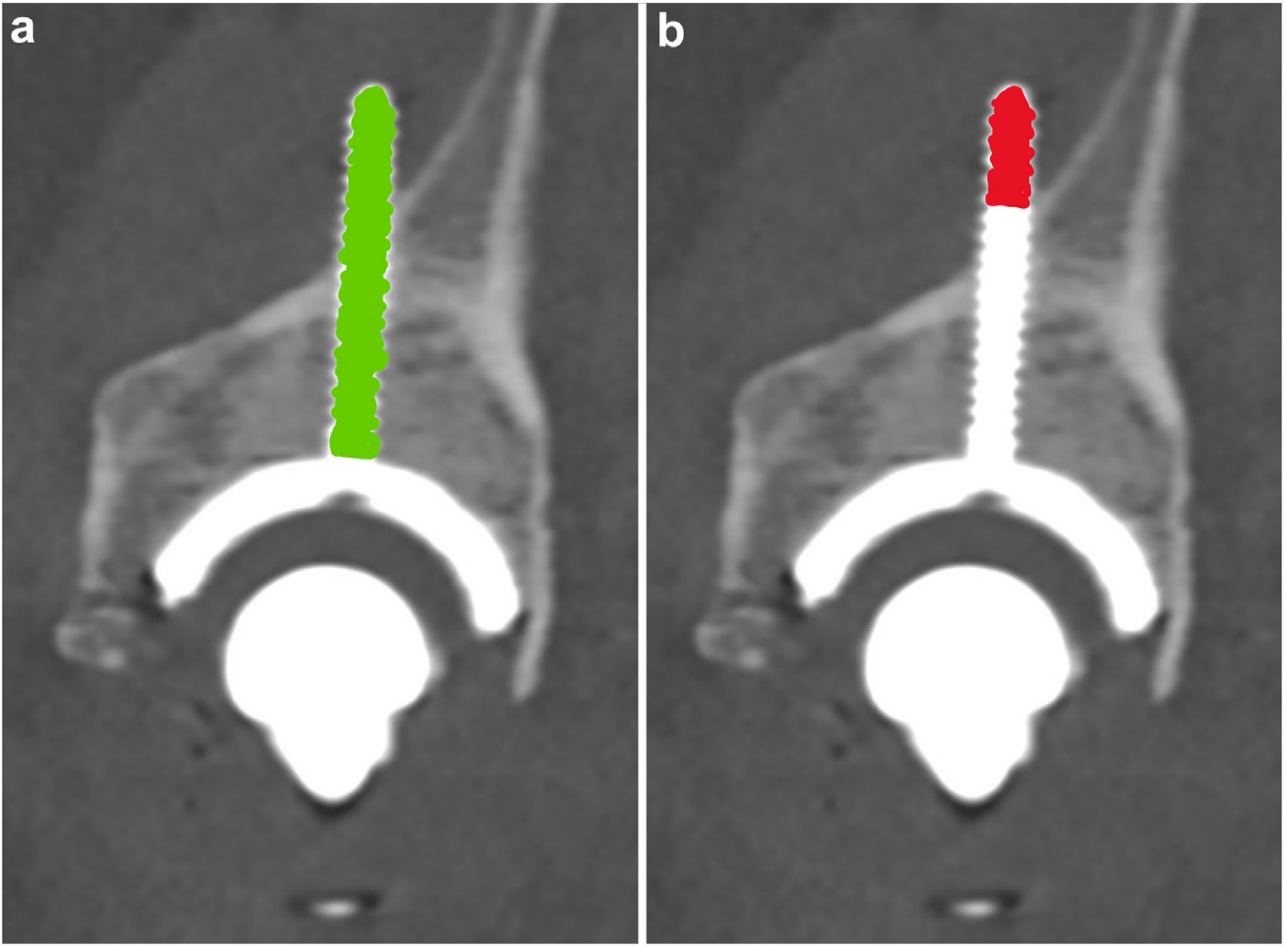
Measurement of screw length in the multiplanar reformation view of a postoperative CT image. Before the measurement of screw length, the 3 planes were aligned along the screw centerline. The *total screw length* (green/a), which is defined as the distance from the outer cup surface to the very tip of the screw, and *screw penetration depth* (red/b), which is defined as the distance from the last screw thread in contact with bone to the screw tip, were measured for each screw.

All evaluations were performed on postoperative CT scans, where a 3D model of the pelvis was created, as described for 3D planning. When a CT scan of the hip was not available after surgery, we used CT scans of the abdomen or pelvis performed for other indications, if appropriate 3D reconstructions could be obtained (*N* = 6). Screw positioning was divided into 3 anatomic regions: the SPR/anterior column, the ischial tuberosity/posterior column, and the ilium (Fig. 4). For each screw, the *number*, *total screw lengths* (Fig. 5a), and *screw penetration depths* (Fig. 5b) were determined separately for each anatomic region.

### Measurement of cup position

**Supplementary Figure 1:**
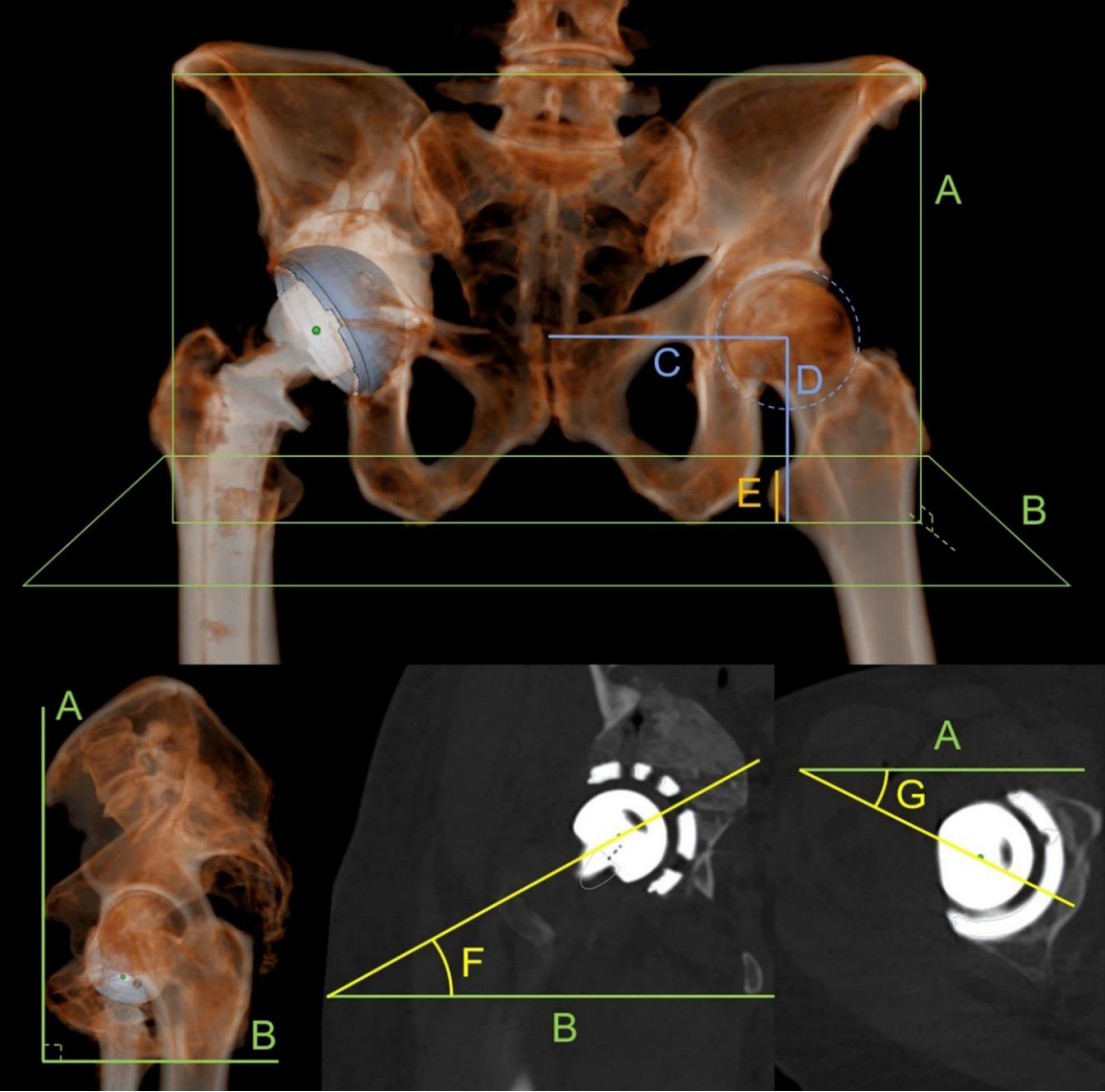
Measuring cup position relies on identifying the anterior pelvic plane (APP; A), defined as the plane to the bilateral anterior iliac spine and the pubis, and the ischial plane (IP; B) defined as the plane to the bilateral inferior ischium and perpendicular to the APP. All distances are measured as the projected distance on the APP. Offset is measured as acetabular offset (C) defined as the distance between the pubic symphysis and the rotational center of the hip, acetabular height (D) defined as the distance between the IP and the rotational center of the hip. Leg length discrepancy is measured as the difference in length between the IP and the minor trochanters (E). Cup inclination (F) is the angle between the cup centerline and the IP. Cup anteversion (G) is the angle between the cup centerline and APP, projected on the IP.

Cup position (*inclination* and *anteversion*), *leg length discrepancy* (LLD), and rotational center (*acetabular offset* and *acetabular height*) were measured in relation to the anterior pelvic plane (defined as the plane between both anterior superior iliac spines and the pubis) and the ischial plane (perpendicular to the anterior pelvic plane running through both ischial tuberosities, Supplementary Fig. 1).

### Inter-rater assessment

All measurements were performed by 2 observers, one orthopedic surgeon (JB) and one product specialist at Sectra Orthopedics AB (RS). Both observers were blinded to the inclusion period and method of planning. An initial set of 10 patients was used to harmonize the measurement methodology. Discrepancies in screw length measurements ≥5 mm, inclination/anteversion ≥5°, and acetabular offset and acetabular height ± 5 mm were resolved by consensus (7 measurements in 7 patients). For all other measurements, the mean for the 2 observers was used in the statistical analysis.

### Follow-up of complications and patient-reported outcome measures

After surgery, the clinical and radiologic follow-ups were individualized based on patient needs and surgeon preference. When the information was available in the patient records (*3D group N* = 17, *2D group N* = 12), hip function was evaluated preoperatively and at 1 year postoperatively using the Merle d’Aubigné-Postel score (a higher score corresponds to a superior outcome) [19,20]. Medical charts were reviewed for complications related to screw placement or penetration.

### Data analysis

The data are shown as means and standard deviations for continuous variables with normal distribution and medians and interquartile range (IQR) for non-normally distributed data.

Comparisons of quantitative variables exhibiting a normal distribution with dichotomous qualitative variables were done with the Students *t*-test. For comparison of quantitative variables with a non-normal distribution, we used the Mann-Whitney *U*-test for dichotomous variables or Kruskal-Wallis test for polychotomous variables. For categorical data, counts and percentages are presented, and a statistical analysis was performed using Person’s chi-squared test. Statistical analyses were performed in IBM SPSS Statistics version 27 (IBM Corporation, Armonk, NY). Statistical significance was set at *P* < .05.

### Ethics and dissemination

The study was approved by the Swedish Ethical Review Authority (Dnr. 2022-04741-02)

## Results

The median *total screw lengths* in the SPR were 16.0 mm in the *2D group* and 25.0 mm in the *3D group* (IQR [12.0-25.0] and [21.2-35.4], *P* = .004). In the SPR, *screw penetration depth* was shorter in the *2D group*, 0.0 mm compared to 1.25 mm in the *3D group* (IQR [0.0-0.5] and [0.0-8.25], *P* = .049). In the *3D group*, screws in the ilium were longer, and *screw penetration depth* was shorter (for details, see Supplementary Table 1). There was no difference between the groups regarding *total screw length* in the ischium (Table 2). The average number of screws utilized was similar in both groups, with medians of 4.5 (IQR [4.0-5.0]) in the *2D group* and 5.0 (IQR [4.75-6.0]) in the *3D group*. Cup inclination and anteversion and acetabular offset did not differ between the groups. Leg length in comparison to the contralateral side was longer in the *3D group* (median, 7.5 mm), and the rotational center of the acetabulum was more distal in the *3D group* (median, 2.5 mm; Table 3). The median Merle d’Aubigné-Postel score increased in both groups; from a preoperative score of 13.0 (IQR [8.0-14.0]) to 17.5 (IQR [15.0-18.0]) at 1 year postoperatively in the *2D group* and from 14.0 (IQR [10.5-15.5]) to 17.0 (IQR [14.0-18.0]) in the *3D group*. No complications due to screw penetration were found in any of the groups.

**Table 2:** Median numbers of screws, total screw lengths, and screw penetration depths per anatomic region in the 2 groups.

Measure and area	2D [IQR]	3D [IQR]	*P*-value
Total number of screws	4.5 [4.0-5.0]	5.0 [4.75-6.0]	.131
SPR	1.0 [1.0-1.0]	1.0 [1.0-1.0]	.712
Ilium	2.5 [1.0-3.0]	3.0 [2.0-4.0]	.141
Ischium	1.0 [1.0-1.0]	1.0 [1.0-1.0]	.908
Total screw length (mm)			
SPR	16.0 [12.0-25.0]	25.0 [21.2-35.4]	.004
Ilium	40.5 [25.5-49.5]	50.5 [30.0-56.0]	.019
Ischium	15.8 [14.2-22.0]	20.0 [15.0-21.0]	.499
Screw penetration depth (mm)			
SPR	0.00 [0.00-0.50]	1.25 [0.00-8.25]	.049
Ilium	1.00 [0.00-6.50]	0.00 [0.00-2.50]	.006
Ischium	0.00 [0.00-2.75]	0.50 [0.00-4.00]	.539

SPR, superior pubic ramus.

**Table 3:** Cup position (inclination and anteversion), position of the rotational center (acetabular height and acetabular offset), and leg length discrepancy compared to the contralateral side.

Measure	2D [IQR]	3D [IQR]	*P*-value
Inclination (°)	47.5 [43.1-52.3]	48.5 [43.0-52.9]	.882
Anteversion (°)	20.3 [13.1-27.4]	21.5 [15.8-25.8]	.721
Acetabular height discrepancy (mm)	−2.8 [−8.6 to 2.6]	+2.5 [0.4-6.1]	.006
Acetabular offset discrepancy (mm)	−1.0 [−3.5 to 2.1]	0.0 [−7.5 to 2.6]	.812
Leg length discrepancy (mm)	+1.0 [−9.3 to 6.0]	+7.5 [0.25-12.6]	.006

The minus sign indicates a higher rotational center, and the plus sign indicates a more distal rotational center than the contralateral side.

## Discussion

With 3D planning, we utilized longer screws for fixation of the cup in the SPR, thereby confirming our hypothesis. We noted deeper penetration of the screw tip through the distal cortex (average, 1.25 mm) compared to the no penetration in the *2D group*. This intentional screw penetration enables purchase in higher-quality cortical bone structures instead of the trabecular bone that becomes weaker with aging [15,21]. The SPR is a suitable region in the pelvis for both cancellous and cortical screw fixation, with the screws appearing to have superior pull-out strength than in other areas in the pelvis [22]. However, important anatomic structures in the vicinity of the SPR have discouraged surgeons from utilizing screws here. We found no adverse effects of penetrating screw tips even when the screws penetrated areas previously described as “danger zones” based on the proximity to vascular structures [8,9,17,23,24]. In contrast to previous cadaver studies, where screw trajectories run perpendicular to the acetabular surface, [8] a modern revision cup allows oblique screw trajectories in the cup, facilitating entry into the bone canal of the SPR. This SPR bone canal is typically utilized by trauma surgeons for *creeping screws* [25] to fix pelvic fractures and has recently been described in silico as suitable for screw fixation in arthroplasty surgery [26]. One prerequisite is the usage of an implant system that allows screw angulation, such as that used in this study. When attempting to place the screw in the bone canal of the SPR, the screw will run roughly parallel to the cortical structure of the SPR, thereby limiting penetration of the soft tissue. To identify the SPR bone canal, we used fenestration of the anterior capsule for direct palpation of the SPR, and in the *3D group*, we used the 3D preoperative planning to guide the surgeon in the surgical field on cup orientation and screw trajectory in relation to bony landmarks such as the SPR. In some cases, a pedicular finder and feeler was used to open the sclerotic bone to enter the SPR and for orientation in the bone canal (Fig. 3a).

The screws in the ilium were roughly 1 cm longer when directed in the standard superior-posterior direction [8]. In these locations, cortical penetration was lower in the *3D group* than that in the *2D group*. The ilium wing contains several possible bone canals for long screws, and the superior bone quality in these areas may make it unnecessary to penetrate cortical structures to ensure screw purchase, if the right screw trajectory can be chosen. It might be that 3D planning contributes to a better understanding of this anatomy, thus increasing the likelihood to place screws without cortical penetration.

The ipsilateral leg was longer than the contralateral side in the *3D group*, due to a combination of a more-distal rotational center of the cup (Table 3) and a longer leg based on the revision femoral component, which is the most-frequent contributor to LLD in total hip arthroplasty [27]. Stem revisions were more frequently performed in the *3D group* (Table 1), which might have contributed to the increase in median LLD. The slightly more distal position of the rotational center of the cup in the *3D group* is required to utilize screw holes in the inferior part of the cup, allowing to enter the SPR, [17] as well as other bone corridors that allow screw fixation in the posterior-inferior direction for increased cup stability [12,28].

While our findings of increased *total screw length* in the *3D group* are clear, we cannot prove a causal relationship between the differences in planning and the placement of longer screws. Similarly, we cannot conclude that longer screws improve mechanical stability. Also, the follow-up was too short, and the sample size too small to evaluate the effect of *total screw length* on the risk of aseptic loosening.

We analyzed *total screw length* in relation to the anatomic region instead of the traditional 4-zone concept based on the risk of damaging vital structures [8] because the 4-zone concept is not precise enough to reflect individual anatomic differences. Screws utilizing the bony anatomy of the individual patient can be long without putting vital structures at risk—we call this the bone canal concept for acetabular cup fixation.

Our study has limitations related to its retrospective nature. As there was no study protocol to guide follow-up, some patients could not be included because of missing postoperative CT and inconsistencies in follow-up. Moreover, patients might have been selected to the 2 different types of preoperative planning based on unidentified selection criteria that might cause confounding results. However, the distribution of acetabular defects was similar between the 2 groups (Table 1).

Despite these limitations, our findings are clear. Standard hemispherical revision shells with the possibility of angular screw placement make it possible to utilize screws in the SPR, an area that was previously considered a danger zone for screw placement in acetabular cups. Our study revealed no complications related to screw insertion or screw tip penetration beyond the far cortex of the SPR. However, it remains critical to handle drills and screws with care in this sensitive anatomical region to prevent damage to adjacent structures.

Based on these findings, we advocate the preoperative identification of the bony anatomy in relation to the implant anatomy in each individual case, rather than the systematic exclusion of certain acetabular quadrants for screw placement, especially in cases where implant stability relies heavily on screw fixation.

## Conclusions

Longer screws were used in the SPR and ilium with preoperative 3D planning. We found no complications related to screw tip penetration beyond the far cortex but we were not able to investigate if longer screws improve fixation. Identifying the bony anatomy in each revision case is important, rather than systematically excluding certain acetabular quadrants.

## Conflicts of interest

Jörg Schilcher is in the speakers’ bureau of or gave paid presentation for Link Sweden, DePuy Synthes, Stryker, and Sectra; is a paid consultant for Link Sweden, Stryker, and Sectra; receives research support as a principal investigator from Link Sweden and Stryker; and is a board member at Swedish Hip and Knee Society. Rolf Scheiderbauer is an employee of and has stock or stock options in Sectra Orthopedics AB. All other authors have no conflicts to disclose.

For full disclosure statements refer to https://doi.org/10.1016/j.artd.2024.101431.

## CRediT authorship contribution statement

**Jonathan Brandt:** Writing – review & editing, Writing – original draft, Validation, Project administration, Investigation, Formal analysis, Data curation. **Rolf Scheiderbauer:** Writing – review & editing, Writing – original draft, Investigation. **Daphne Wezenberg:** Writing – review & editing, Writing – original draft, Formal analysis. **Jörg Schilcher:** Writing – review & editing, Writing – original draft, Visualization, Validation, Supervision, Resources, Project administration, Methodology, Investigation, Funding acquisition, Formal analysis, Data curation, Conceptualization.

## Figures and Tables

**Supplementary Table 1:** Total number of screws categorized by anatomic region and *screw penetration depth* in 5-mm increments for the 2 groups.

Anatomical area	Category	2D (%)	3D (%)	*P*-value
SPR				.172
	<0 mm	15 (71.4)	14 (46.7)	
	0-5 mm	3 (14.3)	5 (16.7)	
	>5 mm	3 (14.3)	11 (36.7)	
Ilium				.031
	<0 mm	26 (49.1)	64 (70.3)	
	0-5 mm	11 (20.8)	14 (15.4)	
	>5 mm	16 (30.2)	13 (14.3)	
Ischium				.396
	<0 mm	13 (59.1)	14 (46.7)	
	0-5 mm	4 (18.2)	11 (36.7)	
	>5 mm	5 (22.7)	5 (16.7)	

SPR, superior pubic ramus.

## Appendix A Supplementary data

Conflict of Interest Statement for Wezenberg

Conflict of Interest Statement for Brandt

Conflict of Interest Statement for Scheiderbauer

Conflict of Interest Statement for Schilcher
