# Supplementary material for: Preoperative Three-Dimensional Planning Using Computed Tomography Improves Screw Placement in Patients Undergoing Acetabular Revision Surgery
Source: Arthroplast Today. 2024 Sep 18;29:101431. doi: 10.1016/j.artd.2024.101431 (PMC11424765; doi:10.1016/j.artd.2024.101431)
Supplement: Conflict of Interest Statement for Brandt [file mmc2.pdf]

INDIVIDUAL CONFLICT OF INTEREST STATEMENT

American Association of Hip and Knee Surgeons

(Adopted from the American Academy of Orthopaedic Surgeons disclosure statement)

The following form **must be filled out completely and submitted by each author (example, 6 authors, 6 forms). All items require a response. If there is no relevant disclosure for a given item, enter "*None*."**

Preoperative three-dimensional planning using computed tomography improves screw placement in patients undergoing acetabular revision surgery

1. Royalties from a company or supplier (The following conflicts were disclosed)
None

2. Speakers bureau/paid presentations for a company or supplier (The following conflicts were disclosed)
None

3A. Paid employee for a company or supplier (The following conflicts were disclosed)
None

3B. Paid consultant for a company or supplier (The following conflicts were disclosed)
None

3C. Unpaid consultants for a company or supplier (The following conflicts were disclosed)
None

4. Stock or stock options in a company or supplier (The following conflicts were disclosed)
None

5. Research support from a company or supplier as a Principal Investigator (The following conflicts were disclosed)
None

6. Other financial or material support from a company or supplier (The following conflicts were disclosed)
None

7. Royalties, financial or material support from publishers (The following conflicts were disclosed)
None

8. Medical/Orthopaedic publications editorial/governing board (The following conflicts were disclosed)
None

9. Board member/committee appointments for a society (The following conflicts were disclosed)
None

Each author must sign AND print or type his/her name, date and submit a separate form

In addition, one BLINDED Conflict of Interest form (no author names used) should be submitted per manuscript with all author disclosures.

Jonathan Brandt	Signed digital – see next page	
Author Name (Print or Type)	Author Signature	Date

Signature page

This document has been electronically signed using eduSign.

eduSign

Electronically signed by
Jonathan Brandt

Date and time of signature
2024-04-14 19:33 UTC

Authenticated by
Linköpings universitet

eduSign

Validate signed documents at: https://validator.edusign.sunet.se

SUNET
