# Supplementary material for: Preoperative Three-Dimensional Planning Using Computed Tomography Improves Screw Placement in Patients Undergoing Acetabular Revision Surgery
Source: Arthroplast Today. 2024 Sep 18;29:101431. doi: 10.1016/j.artd.2024.101431 (PMC11424765; doi:10.1016/j.artd.2024.101431)
Supplement: Conflict of Interest Statement for Schilcher [file mmc4.pdf]

INDIVIDUAL CONFLICT OF INTEREST STATEMENT

American Association of Hip and Knee Surgeons

(Adopted from the American Academy of Orthopaedic Surgeons disclosure statement)

The following form **must be filled out completely and submitted by each author (example, 6 authors, 6 forms). All items require a response. If there is no relevant disclosure for a given item, enter "*None*."**

Preoperative three-dimensional planning using computed tomography improves screw placement in patients undergoing acetabular revision surgery

1. Royalties from a company or supplier (The following conflicts were disclosed)

None

2. Speakers bureau/paid presentations for a company or supplier (The following conflicts were disclosed)

Link Sweden, Depuy Synthes, Stryker, Sectra

3A. Paid employee for a company or supplier (The following conflicts were disclosed)

None

3B. Paid consultant for a company or supplier (The following conflicts were disclosed)

Link Sweden, Stryker, Sectra

3C. Unpaid consultants for a company or supplier (The following conflicts were disclosed)

None

4. Stock or stock options in a company or supplier (The following conflicts were disclosed)

None

5. Research support from a company or supplier as a Principal Investigator (The following conflicts were disclosed)

Link Sweden, Stryker

6. Other financial or material support from a company or supplier (The following conflicts were disclosed)

None

7. Royalties, financial or material support from publishers (The following conflicts were disclosed)

None

8. Medical/Orthopaedic publications editorial/governing board (The following conflicts were disclosed)

None

9. Board member/committee appointments for a society (The following conflicts were disclosed)

Swedish Hip and Knee society

Each author must sign AND print or type his/her name, date and submit a separate form

In addition, one BLINDED Conflict of Interest form (no author names used) should be submitted per manuscript with all author disclosures.

Jörg Schilcher

Signed digital – see next page

Author Name (Print or Type) | Author Signature | Date

Signature page

This document has been electronically signed using eduSign.

eduSign

Electronically signed by
Jörg Schilcher

Date and time of signature
2024-04-14 19:57 UTC

Authenticated by
Linköpings universitet

Validate signed documents at: https://validator.edusign.sunet.se

SUNET
